# Rewiring a secondary metabolite pathway towards itaconic acid production in *Aspergillus niger*

**DOI:** 10.1186/s12934-016-0527-2

**Published:** 2016-07-28

**Authors:** Abeer H. Hossain, An Li, Anja Brickwedde, Lars Wilms, Martien Caspers, Karin Overkamp, Peter J. Punt

**Affiliations:** 1 Dutch DNA Biotech B.V, Utrechtseweg 48, 3704 HE Zeist, The Netherlands; 2 Microbiology and Systems Biology, TNO, Utrechtseweg 48, 3704 HE Zeist, The Netherlands; 3 Molecular Biology and Microbial Food Safety, University of Amsterdam, Science Park 904, 1098 XH Amsterdam, The Netherlands

**Keywords:** Itaconic acid, Secondary metabolite, Controlled-batch cultivation, Citric acid, *citB*

## Abstract

**Background:**

The industrially relevant filamentous fungus *Aspergillus niger* is widely used in industry for its secretion capabilities of enzymes and organic acids. Biotechnologically produced organic acids promise to be an attractive alternative for the chemical industry to replace petrochemicals. Itaconic acid (IA) has been identified as one of the top twelve building block chemicals which have high potential to be produced by biotechnological means. The IA biosynthesis cluster (*cadA, mttA* and *mfsA*) has been elucidated in its natural producer *Aspergillus terreus* and transferred to *A. niger* to enable IA production. Here we report the rewiring of a secondary metabolite pathway towards further improved IA production through the overexpression of a putative cytosolic citrate synthase *citB* in a *A. niger* strain carrying the IA biosynthesis cluster.

**Results:**

We have previously shown that expression of *cadA* from *A. terreus* results in itaconic acid production in *A. niger* AB1.13, albeit at low levels. This low-level production is boosted fivefold by the overexpression of *mttA* and *mfsA* in itaconic acid producing AB1.13 CAD background strains. Controlled batch cultivations with AB1.13 CAD + MFS + MTT strains showed increased production of itaconic acid compared with AB1.13 CAD strain. Moreover, preliminary RNA-Seq analysis of an itaconic acid producing AB1.13 CAD strain has led to the identification of the putative cytosolic citrate synthase *citB* which was induced in an IA producing strain. We have overexpressed *citB* in a AB1.13 CAD + MFS + MTT strain and by doing so hypothesize to have targeted itaconic acid production to the cytosolic compartment. By overexpressing *citB* in AB1.13 CAD + MFS + MTT strains in controlled batch cultivations we have achieved highly increased titers of up to 26.2 g/L IA with a productivity of 0.35 g/L/h while no CA was produced.

**Conclusions:**

Expression of the IA biosynthesis cluster in *Aspergillus niger* AB1.13 strain enables IA production. Moreover, in the AB1.13 CAD strain IA production resulted in overexpression of a putative cytosolic citrate synthase *citB*. Upon overexpression of *citB* we have achieved titers of up to 26.2 g/L IA with a productivity of 0.35 g/L/h in controlled batch cultivations. By overexpressing *citB* we have also diminished side product formation and optimized the production pathway towards IA.

**Electronic supplementary material:**

The online version of this article (doi:10.1186/s12934-016-0527-2) contains supplementary material, which is available to authorized users.

## Background

**Fig. 1:**
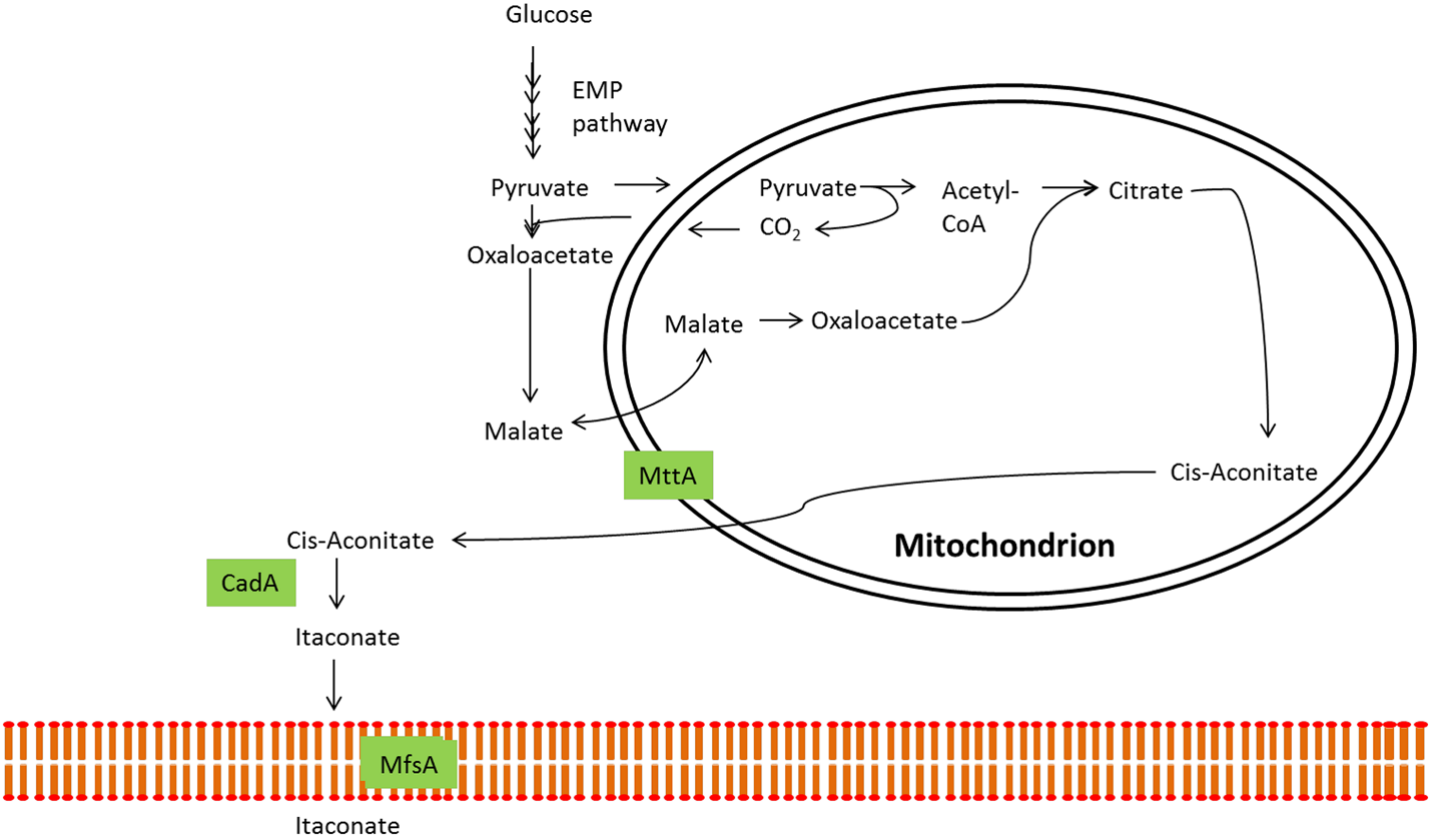
Itaconic acid production pathway in *Aspergillus terreus.* The itaconic acid biosynthesis pathway in *Aspergillus terreus*. Glucose is converted to pyruvate through glycolysis. Pyruvate is converted to acetyl-CoA that together with oxaloacetate forms citric acid and start the TCA cycle. MttA transports* cis*-aconitate from the mitochondrion into the cytosol where CadA facilitates the conversion of* cis*-aconitic acid to itaconic acid and concomitantly MfsA transports itaconic acid out of the cell. Note that only direct pathway steps towards IA production are shown

Global carbon emissions as a result of petroleum-based processing and products are forcing industries to look for alternative processing and production methods. Bio-based organic acid production promises to be an attractive alternative for the chemicals industry to replace petrochemicals and for the food industry as an food additive. For thousands of years humans have employed the help of microorganisms for the production of organic acids e.g. lactic acid and acetic acid [1]. More recently, filamentous fungi have stepped forward as potent producers of organic acids and the industrial producers of enzymes. Itaconic acid (IA) is a C-5 dicarboxylic acid that has been recognized by the US Department of Energy as one of the top 12 building block chemicals with high potential to be produced using biotechnology [2]. IA has a broad application potential in the chemicals industry as co-monomer in the production of polymers, surfactants and fibers (for review see [3]). Another application potential is in agriculture or medicine as a bioactive compound [4]. Since the 1960s, industrial production is facilitated by its natural producer *Aspergillus terreus*, a filamentous fungus, at a maximum production rate of 1.2 g/L/h and fermentations can reach a final titer of 86.2 g/L [5]. Although higher IA levels have been reported in literature these have been reached under conditions that are not suitable for industrial application [6, 7]. Li et al. have previously proposed *Aspergillus niger* as a suitable production host for the industrial production of IA due to the hosts optimized pathways towards organic acids. Therefore, these researchers have successfully identified the IA biosynthesis cluster in *Aspergillus terreus* by using a clone based transcriptomics approach. This gene cluster consists of the* cis*-aconitate decarboxylase (*cadA*), mitochondrial tricarboxylate transporter (*mttA*) and the major facilitator superfamily protein (*mfsA*) (Fig. 1) [8]. Expression of *cadA* in *A. niger* resulted in IA production albeit at low levels [8–10]. This low production was increased by expressing *mfsA* and *mttA* in an IA producing *A. niger* transformant (AB1.13 CAD 4.1). The resulting AB1.13 CAD + MFS3.9 and AB1.13 CAD + MTT 1.4 improved production by twofold [11]. In this research we have continued this work towards third generation IA production *A. niger* strains. In a first approach we reconstituted strains with the complete gene cluster. To identify other genes relevant for improving IA production in *A. niger* we have performed a preliminary RNA-Seq analysis to identify genes differentially expressed in relation to IA production. One of the identified genes encodes a putative cytosolic citrate synthase, of which overexpression resulted in improved IA production.

## Methods

### Strains, vector construction and transformation methods

All strains used in this study have an AB1.13 background [12] and are listed in Table 1. All strains are stored in 30 % glycerol at −80 °C and maintained on potato dextrose agar plates (BD, New Jersey, USA) or on agar containing minimal medium plates (16 g/L agar, 6 g/L NaNO_3_, 0.52 g/L KCl, 1.52 g/L KH_2_PO_4_, 10 g/L glucose, 0.0022 g/L ZnSO_4_ × 7H_2_O, 0.0011 g/L H_3_BO_3_, 0.0005 g/L MnCl_2_ × 4H_2_O, 0.0005 g/L FeSO_4_ × 7H_2_O, 0.00017 g/L CoCl_2_ × 6H_2_O, 0.00016 g/L CuSO_4_ × 5H_2_O, 0.00015 g/L NaMoO_4_ × 2H_2_O, 0.005 g/L Na_2_EDTA and 0.5 g/L Mg_2_SO_4_). Spore suspensions were prepared using physiological salt solution and stored at 4 °C for up to 1 year.

**Table 1:** List of strains used during this study

Strain/transformant	Abbreviation	Strain description
AB1.13 CAD 4.1	CAD 4.1	Selected pyrG + transformant of cadA expressing transformant (CAD10.1) of AB1.13: [13]
AB1.13 CAD + MFS 3.9	MFS 3.9	Selected mfsA expressing transformant of CAD10.1: [11]
AB1.13 AD + MFS + MTT #08; #49; #63	AB1.13 #08; AB1.13 #49; AB1.13 #63	Selected *mttA* expressing transformants of AB1.13 CAD + MFS 3.9
AB1.13 CAD + MFS + MTT + CitB #77; #99; #101; #113	CitB #77; CitB #99; CitB #101; CitB #113	Selected *citB* overexpressing transformants of AB1.13 CAD + MFS + MTT #49

Transformations with *mttA* were performed using AB1.13 CAD + MFS 3.9 strain and *citB* was overexpressed in the resulting AB1.13 CAD + MFS + MTT #49 strain. The *mttA* gene construct was the same as used in the previous study of Li et al. [11] and introduced by co-transformation with the pAN-7 plasmid, that contains the *hph* resistance gene, in an ratio of 1:10 (10 µg construct:1 µg marker). The transformed protoplasts were plated on MM agar plates containing sorbitol and Hygromycin B (15 g/L agar, 6 g/L NaNO_3_, 0.52 g/L KCl, 1.52 g/L KH_2_PO_4_, 1 % glucose, 0.5 g/L MgSO_4_, 0.022 g/L ZnSO_4_·7H_2_O, 0.011 g/L H_3_BO_3_, 0.005 g/L MnCl_2_·4H_2_O, 0.005 g/L FeSO_4_·7H_2_O, 0.0017 g/L CoCl_2_·6H_2_O, 0.0016 g/L CuSO_4_·5H_2_O, 0.0015 g/L NaMoO_4_·2H_2_O, 0.05 g/L Na_2_EDTA, 218.6 g/L sorbitol and 200 mg/L hygromycin B) and incubated at 33 °C for up to 1 week until colonies were visible.

To establish overexpression of *citB* a PCR amplified copy of this gene was generated with the primers citB-F3-ATG + *Bsm*BI (5′-CGTCTCCCATGCCCGACATCGCATCCAAC-3′) and citB-R1 + *Nco*I (3′-ATCCGTCAAAGCGAGAGTGGTACC-5′) using genomic *Aspergillus niger* DNA as template. The resulting PCR fragment was digested with* Bsm*BI/*Nco*I and the fragment was inserted into an pABgpd1 containing the *A. niger gpdA* expression signals thus establishing the *citB* expression vector pAB*gpd*I-*citB* [14]. This construct was co-transformed with pAN8-1, that harbours the phleomycin resistance marker [15], in an ratio of 1:10 (1 µg marker: 10 µg construct). Transformed protoplasts were plated on MM agar plates containing sorbitol and phleomycin (50 mg/L).

### Fermentations

Controlled batch-cultivations were performed on 5 l scale benchtop New Brunswick Scientific fermenters (BioFlo 3000) at 33 °C. Starting pH was 3.5 after inoculation and medium was allowed to naturally acidify till pH 2.3 and then kept at pH 2.3 by addition of 4 M KOH. Dissolved oxygen (DO) tension was 25 % at moment of inoculation and DO dropped till 20 % and kept at 20 %. The system was calibrated with 100 % sterile air as 100 % DO and 100 % N_2_ as 0 % DO. The fermenter was inoculated by 72 h old 100 ml baffled shake flask cultures containing 1.0 × 10^8^ spores. Medium composition for fermentation and pre-culture (M12 + Cu) are listed in Table 2 [13].

**Table 2:** Composition of medium 12 + Cu, which is used as production medium for IA (Adapted from Li et al. [13])

Component	Final concentration (g/L)
NH_4_SO_4_ [(NH_4_)_2_SO_4_]	2.36
KH_2_PO_4_	0.11
MgSO_4_ × 7H_2_O	0.5
CuSO_4_ × 5H_2_O	0.005
FeIISO_4_ × 7H_2_O	0.0006
ZnSO_4_ × 7H_2_O	0.0006
NaCl	0.074
CaCl_2_ × 2H_2_O	0.13
Glucose	100

### Colony PCR

Successful integration of *mttA* constructs was determined with colony PCR [16].

### HPLC

Metabolite analysis was performed using a WATERS e2695 separations module equipped with an Aminex HPX-87H column (Bio-Rad) and 5 mM H_2_SO_4_ as eluent. Detection of peaks occurred simultaneously by a refractive index detector (WATERS 2414) and a dual-wavelength detector (WATERS UV/Vis 2489). Data processing was done with Empower Pro software (Empower 2 Software, copyright 2005–2008, Waters Corporation, Milford, Massachusetts, USA).

### Microplate based transformant screening

Plates carrying transformed cells were allowed to grow and sporulate for 1–2 weeks after which individual colonies were transferred to a selective MM plate. Individual colonies from this plate were each streaked on a separate selective MM plate to isolate single colonies that in turn was used to inoculate a 1 mL liquid culture in a 96-wells deepwell plate containing M12 + Cu. This 96-wells plate was incubated for 72 h at 33 °C and 850 RPM. Supernatant was filtered over a 0.22 µM filter and analyzed on the HPLC for IA production. Colonies that showed promising results in terms of IA production were grown on plates to prepare spore suspensions.

### Southern blotting

Shake flask (500 mL) cultures containing 100 mL M12 + Cu were inoculated with 1.0 × 10^7^ spores and grown for 3 days at 33 °C and 125 RPM. Upon harvesting the mycelia were washed with demi water and immediately frozen in liquid nitrogen. The frozen mycelia were ground to a fine powder using a sterile mortar and pestle. Three hundred microgram of ground mycelia were sampled per strain and DNA isolation occurred using the mag kits from LGC (LGC, Queens Road, Teddington, Middlesex, TW11, 0LY, UK) and per manufacturers instruction. Quality of isolated DNA was tested by running on 0.8 % agarose gel. Four microgram genomic DNA was digested per strain with* Bgl*II and* Mlu*I (New England Biolabs) and run on agarose gel o/n. Blotting occurred on Nitrocellulose N+ blotting paper (Amersham Biosciences) using SSC (175.5 g NaCl and 88.2 g Na-Citrate in 1 L; 20× stock) solution. After blotting the membranes were treated in UV-chamber to cross-link DNA. Labeling and detection were performed using the DIG Easy Hyb labeling and detection kit for Southern blotting purposes (Roche Life Sciences) per the manufacturers instructions.

### IA toxicity assay

Shakeflasks (500 mL) were prepared with 100 mL M12 + Cu supplemented with the following IA concentrations: 0, 10, 20, 30 and 40 g/L. pH was adjusted to 2.3 by addition of KOH/H_2_SO_4_. The flasks were inoculated with 1 ml of overnight grown pre-culture. After 7 days the mycelium was harvested and the biomass was determined by measuring the dry weight.

### RNA isolation and transcriptome analyses

Two 500 mL baffled shakeflasks containing 100 mL M12 + Cu were inoculated with 1.0 × 10^6^ spores/ml of AB1.13 WT and CAD 4.1 and incubated at 33 °C and 125 RPM for 48 h. After cultivation the mycelium was harvested, washed with distilled water and frozen in liquid N_2_. The mycelium was ground with mortar and pestle to a fine powder and RNA extraction proceeded using the Trizol method. Quality control was checked on 1× MOPS/6 % Formaldehyde agarose gels and stained with ethidium bromide.

Service XS in Leiden, NL performed digital gene expression profiling experiments based on RNA-Seq with an Illumina HiSeq 2000 System. Approximately 8–32 M unfiltered paired-end (PE) reads (99 bp/read on ~320 bp cDNA inserts) were obtained. Reads were trimmed of the first two bases of the 5′ end because these bases showed an aberrantly low GC content. The reads were then further filtered, such that all quality phred scores after filtering are at least 22, with a read-length of at least 40 bases. Around 70–80 % of the bases passed these criteria (including a 2 % loss because of clipping). After filtering the #PE-reads/samples were 10.9 and 10.0 M for the CAD and WT sample respectively.

Reads were aligned to the 20 contigs in a FastA file of the *Aspergillus niger* reference genome (from http://www.ebi.ac.uk/ena). Source EMBL annotations were converted to GFF format. The EMBL data appeared to be derived from multiple sources with different feature tags. These were converted to one uniform GFF format that could be accepted by our third-party software (consistent gene_ids across all contigs). Missing gene definitions (e.g. for CAD) were inserted. The reads were aligned to the reference genome using software based on a Burrows-Wheeler Transform (BWT) algorithm. A mismatch rate of 4 % was allowed for the alignment. The maximum insertion length was 3. The maximum deletion length was 3. All samples had more than 85 % of the reads aligned, resulting in SAM alignment files. Gene expression was measured as the number of aligned reads to reference genes and was normalized to RPKM values (Reads per kb per million reads; Mortazavi et al. [17]). Full analysis will be published elsewhere (Hossain et al. manuscript in preparation).

## Results

### Expression of IA biosynthesis cluster

Based on previous results we established in our research that expression of the putative dicarboxylic acid exporter MfsA and a mitochondrial transporter MttA, both encoded by genes in the IA gene cluster in an *A. niger* strain carrying the* cis*-aconitate decarboxylase, resulted in increased IA levels [11]. The positive effect of overexpression of the dicarboxylic acid transporter was also confirmed in *A. terreus* [18]. Interestingly, different results were obtained in similar research recently published by van der Straat et al. [9]. More detailed analysis of the data revealed that in the latter research, a codon-optimized gene copy was used, based on an incorrectly annotated gene model, resulting in an unspliced intron in the gene sequence used (Additional file 1), explaining why expression of this gene copy did not result in increased IA secretion.

**Fig. 2:**
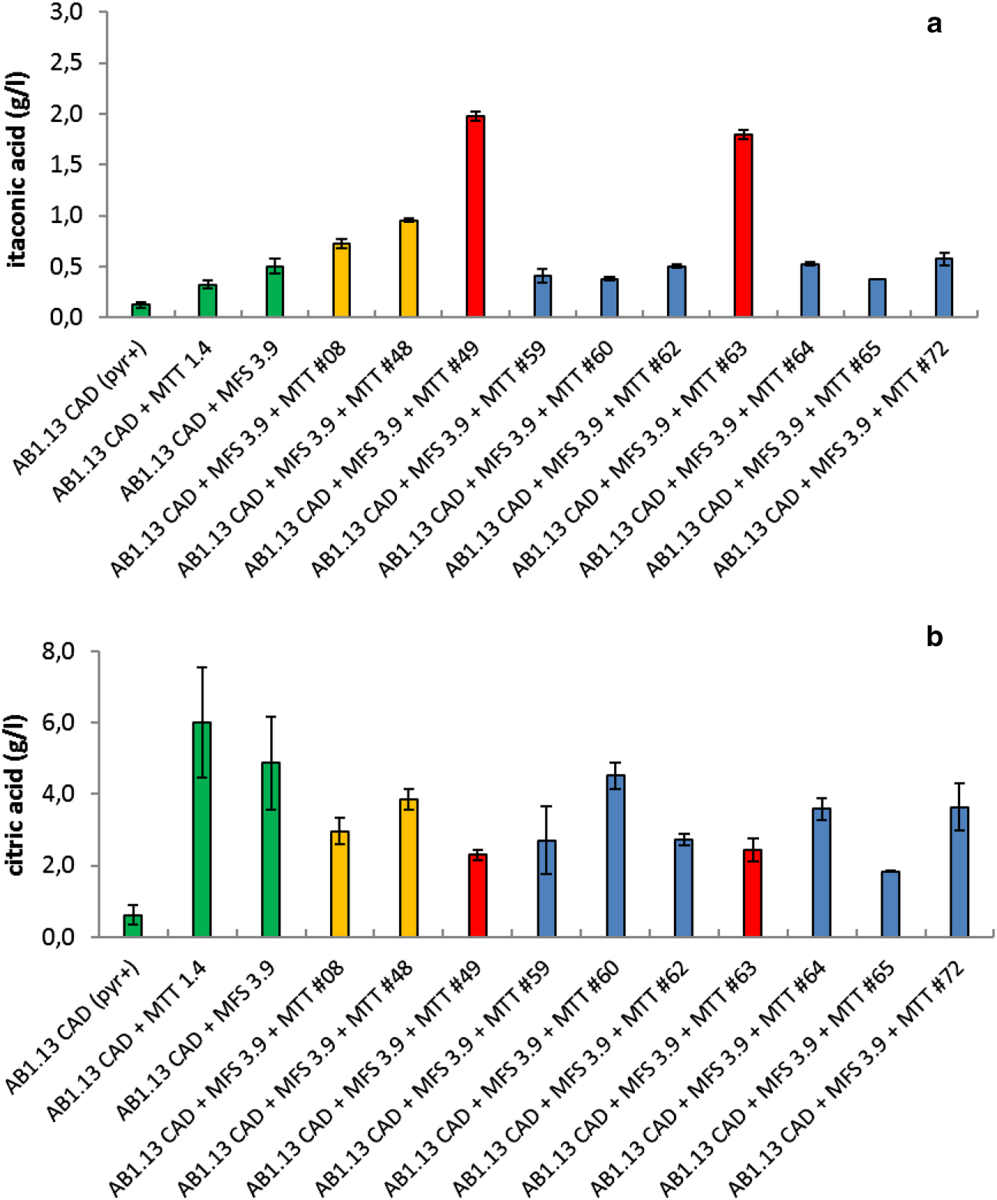
Shake flask cultivation of itaconic acid producing *A. niger* transformants. HPLC analysis of shake flask experiment with AB1.13 itaconic acid producing transformants. Shake flasks were cultivated for 5 days prior to analysis. All strains were analyzed in triplicate. **a** Production of itaconic acid is compared amongst strains carrying CAD, CAD + MFS, CAD + MTT and CAD + MFS + MTT. Strains AB1.13 #49 and #63 have an fivefold improvement in IA production compared with the parental strain AB1.13 MFS 3.9. **b** Production of citric acid is compared amongst strains carrying CAD, CAD + MFS, CAD + MTT and CAD + MFS + MTT. The highest IA producers AB1.13 #49 and #63 produce the lowest amount of CA among all strains carrying the full IA biosynthesis cluster

**Fig. 3:**
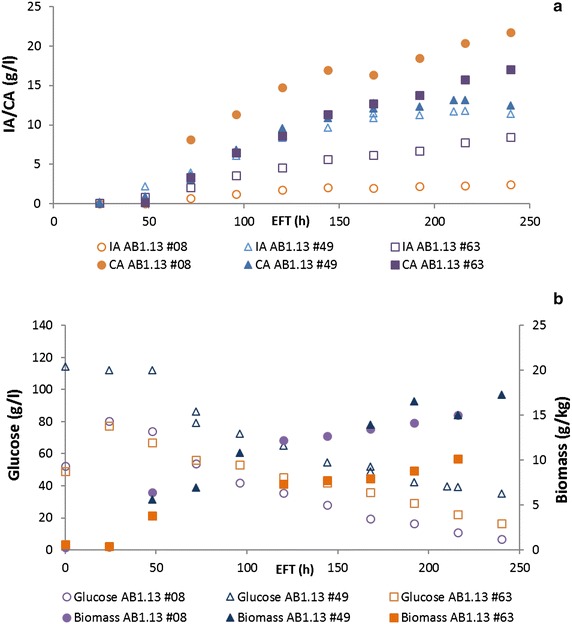
Controlled batch cultivation of itaconic acid producing *A. niger* transformants. Controlled batch cultivation of AB1.13 strains carrying the full IA biosynthesis cluster. **a** HPLC analysis of organic acid production during controlled batch cultivations of AB1.13 strains #08, #49 and #63. The highest IA titer is achieved by AB1.13 #49 (11.74 g/L). AB1.13 #08 produces the most CA (21.99 g/L) from all three strains and achieves the lowest IA titer (2.48 g/L). AB1.13 #63 produces 9.52 g/L IA and 18.03 g/L CA. **b** Biomass development and glucose consumption during controlled batch cultivations of strains #08, #49 and #63

In our previous results we analyzed a small number of strains that could carry all three genes of the IA gene cluster. However, none of the strains analyzed showed increased IA levels compared to the parental strain containing only the *cadA* plus *mttA* gene [11]. In a further attempt to generate *A. niger* strains with all three genes of the cluster we now introduced *mttA* gene copies in strain AB1.13 CAD + MFS 3.9, carrying *cadA* and *mfsA* [11]. Presence of *mttA* gene was confirmed in 68 out of 92 transformants by performing colony PCR. Based on the results obtained during microtiter plate screening in many of the transformants increased IA levels were observed (data not shown). From a selection of these 68 transformants ten strains were selected for further analysis in shake flasks. As shown in Fig. 2a and b apparently different classes of transformants were obtained. Those that perform at the same level as the parental strain (blue bars), those that perform 1.5–2.0× better than the parental strain (yellow bars) and mutants that perform 5× better than the parental strain (red bars). Citric acid (CA) production increases when either *mfsA or mttA* genes are expressed separately in a *cadA* background. But when both genes are expressed simultaneously CA production decreases again. Interestingly, the highest producers of IA AB1.13 CAD + MFS + MTT #49 and #63 strains are amongst the lowest CA producing transformants. For further analysis two strains with the highest level of IA were selected (AB1.13 #49 and #63) alongside with the AB1.13 #08 strain which showed intermediate improvement in IA production (Fig. 2a yellow bars). As shown in Fig. 3a also in controlled batch cultivation the AB1.13 #49 and #63 strains showed clearly increased IA titers, productivity and yield compared to the AB1.13 #08 and the parental AB1.13 CAD + MFS 3.9 strain [11], which produced comparable levels (Table 4). In the controlled batch cultivation AB1.13 #49 showed a superior IA titer (11.72 g/L) compared to the #63 strain (9.52 g/L). Also the productivity of #49 (0.08 g/L/h) and yield (0.16 g/g) are higher than the #63 strain (0.05 g/L/h and 0.13 g/g). The levels of CA produced by strain #49 (13.17 g/L) and #63 (18.02 g/L) are an indicator that there is ample precursor available for conversion to IA and thus improvement of IA production.

### IA toxicity

The obtained IA levels in these transformants are quite promising, but for commercial application considerably higher titers and yields would be required. As shown in Fig. 3b, during fermentation of the two high IA producing strains less glucose is consumed compared to the lower IA producer (AB1.13 #08), whereas biomass and product formation ceased. As this retarded growth could be a consequence of the fact that IA is regarded to be mildly anti-microbial [4], the effect of IA on the growth of *A. niger* was tested.

**Fig. 4:**
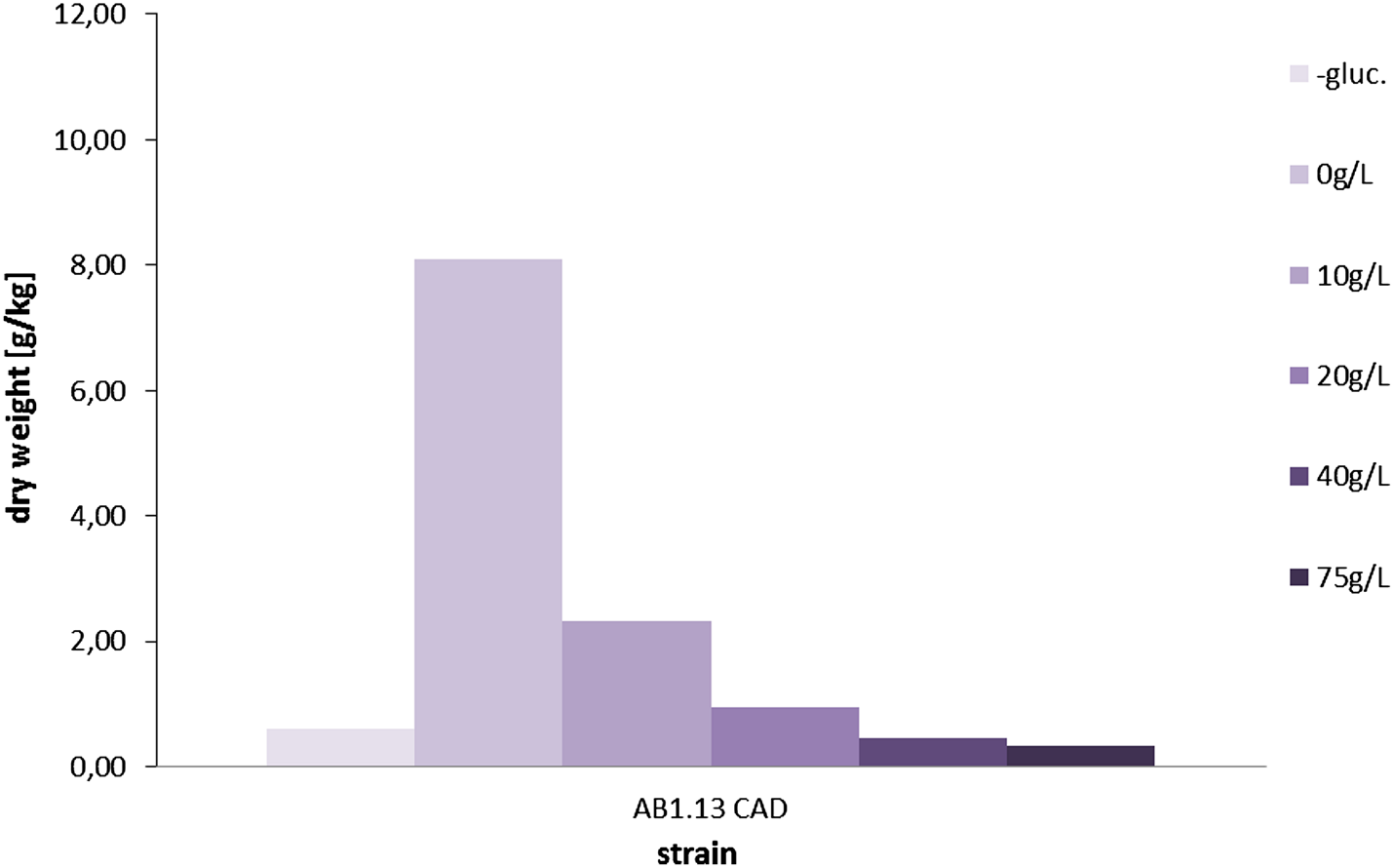
Growth evaluation of *A. niger* in the presence of extracellularly added itaconic acid. Growth of AB1.13 CAD strain in M12 + Cu supplemented with several concentrations of itaconic acid. Medium devoid of C-source is used as negative control. An extracellular concentration of 10 g/L IA in the production medium results in an 75 % decrease in biomass development. At 20 g/L IA growth is at sub-inhibitory level and even higher concentrations of IA further deteriorate growth

As shown in Fig. 4 the growth of *A. niger* is already hampered at concentrations of 10 g/L IA in the extracellular medium. At even higher concentrations (20, 40 and 75 g/L) the detrimental effects of IA on growth become more evident. Based on this result we hypothesize that IA toxicity may indeed limit its production in *A. niger*.

### Transcriptome analysis of an IA producing *A. niger* strain using RNA-Seq

Increased levels of IA in the culture medium have shown a clear growth effect (Figs. 3, 4). Apparently, production of this novel metabolite in *A. niger* could result in considerable effects on fungal metabolism. Therefore we have performed a preliminary transcriptome analysis comparing mRNA samples from two day old shake flask cultures of the wild-type *A. niger* AB1.13 and low IA producing strain CAD 4.1. Two technical replicate samples of each strain were sequenced and differential expression analysis of the RNA sequence data was carried out to identify those organic acid pathway genes showing increased or decreased expression in the CAD 4.1 strain. In Additional file 1 the expression levels of all genes with a potential role in organic acid biosynthesis pathway are shown. The genes were selected based on extensive sequence homology of the encoded proteins with experimentally validated *S. cerevisiae* counterparts [19]. As shown in this table, in *A. niger* multiple genes encoding enzymes with putative citrate synthase activity are present. In silico predictions indicate that different members of this protein family are targeted to different cellular compartments. Our expression data suggest that in the WT strain these activities are mainly expressed from genes encoding mitochondrial protein, whilst in the CAD 4.1 strain genes encoding cytosolic proteins are expressed. It is evident that a gene encoding a putative cytosolic citrate synthase (*citB*) [20, 21] is induced in the CAD 4.1 strain.

Upon closer examination, the *citB* gene is tightly clustered and part of a secondary metabolite cluster that consists of genes encoding the subunits of a fatty acid synthase, a regulation gene, a major facilitator transporter, a cytosolic *cadA*-like protein and a dienelactone hydrolase that are all induced together in the CAD 4.1 strain (Additional file 1). Based on these results we propose that this gene cluster encode genes constituting a biosynthetic pathway for a metabolite which is a fatty acid derived organic acid. Interestingly, literature research on *A.niger* secondary metabolites [22] reveal that *A*. *niger* is capable of producing alkyl-itaconic acids, also known as tensyuic acid [23].

**Fig. 5:**
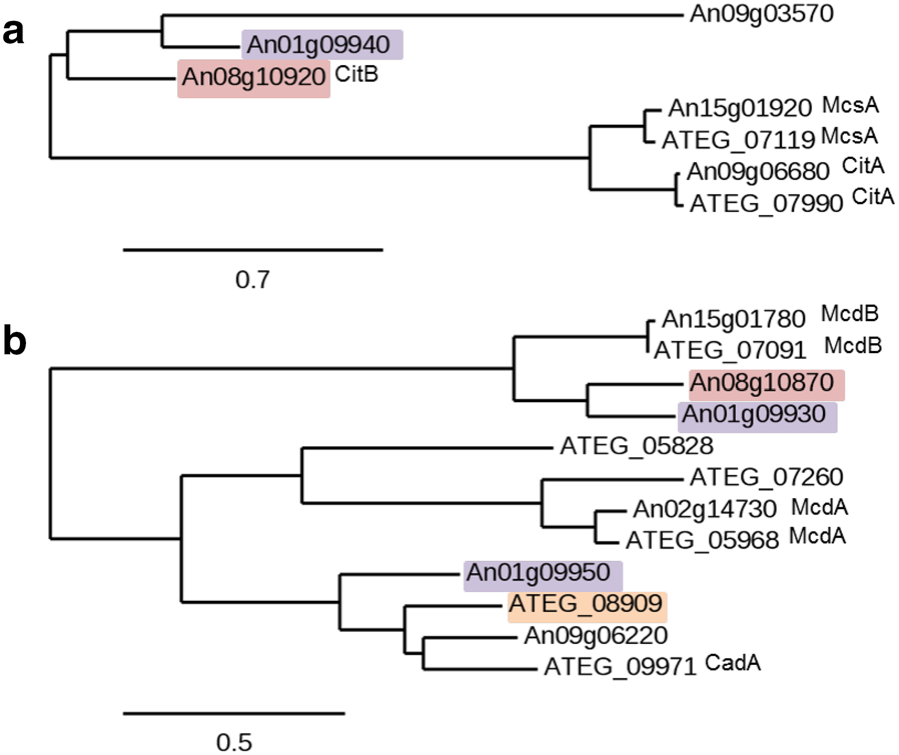
Phylogenetic trees of selected pathway enzymes in *A. niger* and their orthologues in *A. terreus.* Phylogenetic trees of amino acid sequences of selected pathway enzymes in *A. niger* and their orthologues in *A. terreus*. The encoded *Aspergillus niger* proteins are designated with their corresponding An numbers (Anxxgxxxxx) and *Aspergillus terreus* proteins are designated with their corresponding ATEG numbers (ATEG_xxxxx). Proteins that are clustered together are shaded in the same colour throughout the separate classes. Phylogenetic trees were constructed using the phylogeny.fr online tool [38]. **a** Phylogenetic tree of known citrate synthase proteins and their orthologues in *A. niger* and *A. terreus*. Interestingly, the citrate synthase proteins from *A. niger* without predicted mitochondrial localization are clustered separately and no known orthologues are present in *A. terreus*. **b** Phylogenetic tree of CadA and predicted CadA-like enzymes in *A. niger* and *A. terreus*

Further analysis of the *citB* gene sequence revealed that unlike the canonical citrate synthase *citA*, the encoded CitB protein contains no predicted mitochondrial or other targeting sequence and therefore is putatively targeted to the cytosol. Additional file 1 shows sequence comparison of the canonical CitA protein with the CitB protein that appears to show only 22 % sequence similarity. More detailed phylogenetic analysis reveals that the proteins encoded by *citA* and *citB* are only distantly related to each other, quite possibly indicating different roles in metabolism (Fig. 5a). Interestingly, BLAST searches indicate that CitB orthologues are only present in a limited number of fungal strains. Of particular interest is that in *A. terreus*, from which the IA gene cluster originates, no CitB orthologue is present (Fig. 5a). Upon aligning the protein sequences of the PrpD-like proteins of *A.niger* with its orthologues in *A. terreus* it became clear that CadA and the upregulated CadA-like protein (An08g10870) are only distantly related (Fig. 5b). Upon closer examination of enzymes necessary for IA synthesis other than citrate synthase, i.e. aconitase and* cis*-aconitate decarboxylase, we found that only the *A*. *nigeracoA* (An08g10530) gene is slightly upregulated in the CAD 4.1 strain (Additional file 1).

More recently Geiser et al. have reported an alternative IA biosynthetic pathway in the smut fungus *Ustilago maydis* [24]. In this organism IA is produced via the unusual metabolite trans-aconitate which is converted to IA. Genes homologous to the two *U. maydis* genes involved in this alternative IA biosynthetic pathway *tad1* and *adi1* are also present in *A. niger* (Additional file 1), however as shown in this table neither in the WT nor in the CAD 4.1 strain any of these genes is strongly expressed, indicating that this pathway is not actively used in *Aspergillus niger*.

### CitB overexpression

**Fig. 6:**
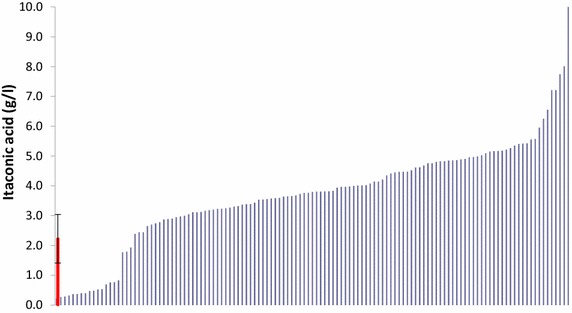
Microtiter plate cultivation of *citB* overexpressing mutants. HPLC results from a five-day old microtiter plate culture of *citB* overexpressing mutants of AB1.13 CAD + MFS + MTT #49.The parental control strain is indicated by a *red bar* with standard deviation. Out of 125 transformant colonies that were tested, 106 colonies produced more IA than the average produced by the parental strain

Based on the results obtained from the transcriptome analysis it was considered that overexpression of *citB* could potentially facilitate the formation of citrate in the cytosol where it could be further processed into IA by the CadA enzyme. Therefore, a *citB* expression vector was designed and introduced in AB1.13 CAD + MFS + MTT #49, being our best IA producer. In total, 129 colonies were identified on phleomycin containing selection plates as potential *citB* overexpressing transformants that were further analyzed in a microtiter plate screen. In the microtiter plate screen many of the obtained transformants produced more IA than the parental strain indicating a role of *citB* in increasing IA production in strains carrying the full IA biosynthesis cluster (Fig. 6). Based on the screening results a number of strains with more than twofold increased IA levels were selected to be analyzed by Southern analysis to confirm the presence and number of additional *citB* gene copies as well as to establish the number of stably integrated *cadA*, *mfsA* and *mttA* gene copies (Table 3). Four of the selected *citB* overexpressing strains were used for further research under controlled fermentation conditions.

**Table 3:** Relative copy numbers of introduced genes

Strain	Southern blot results (rel. no. copies)			
	*cadA*	*mfsA*	*mttA*	*citB*
AB1.13 CAD 4.1 pyrG+	8	0	0	1
AB1.13 CAD + MFS 3.9	8	2	0	1
AB1.13 CAD + MTT 1.4	8	0	1–2	1
AB1.13 CAD + MFS + MTT #49	8	2	1	1
AB1.13 CAD + MFS + MTT #63	8	2	0	1
AB1.13 CAD + MFS + MTT #08	8	2	1–3	1
AB1.13 CAD + MFS + MTT + CitB #99	8	2	1	10
AB1.13 CAD + MFS + MTT + CitB #113	8	2	1	3
AB1.13 CAD + MFS + MTT + CitB #77	8	2	1	7
AB1.13 CAD + MFS + MTT + CitB #101	8	2	1	3

### Controlled batch-cultivations

**Fig. 7:**
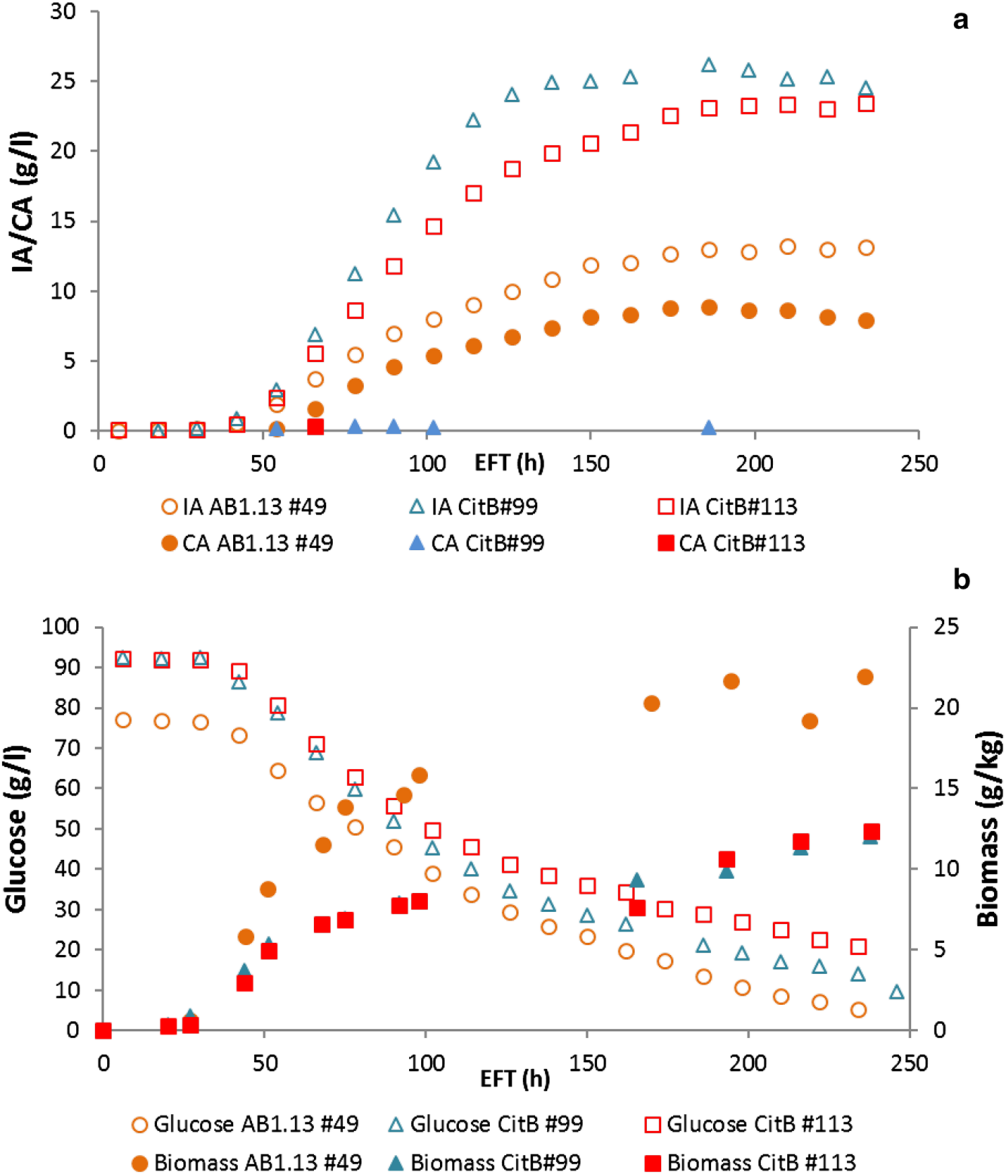
Controlled batch cultivations of *citB* overexpressing mutants and parental strain. **a** HPLC analysis of controlled batch cultivations of AB1.13 CitB mutants #99, 113# and AB1.13 CAD + MFS + MTT #49. Both *citB* transformants show complete conversion of CA into IA as there is little to no production of CA by these strains. IA production halts after 120 h for CitB #99 and after 192 h for CitB #113. The highest titer reached by the parental AB1.13 #49 strain is 12.96 g/L IA and 8.91 g/L CA whereas CitB #99 and #113 reach a highest titer of 26.2 and 23.42 g/L IA and 0.3 and 0.35 g/L CA. **b** Biomass development during controlled batch cultivations of AB1.13 CitB#99, #113 and AB1.13 CAD + MFS + MTT #49. Glucose is consumed at identical rate by all three strains but biomass formation is much lower for the *citB* overexpressing strains #99 and #113. CitB #99 and #113 reach a final biomass concentration of 12.0 and 12.38 g/kg, whereas the parental AB1.13 #49 strain reaches a final biomass concentration of 21.96 g/kg. Also it becomes evident that fungal growth is severely hampered by relatively high amounts of IA in the fermentation broth of CitB#99 and #113

**Fig. 8:**
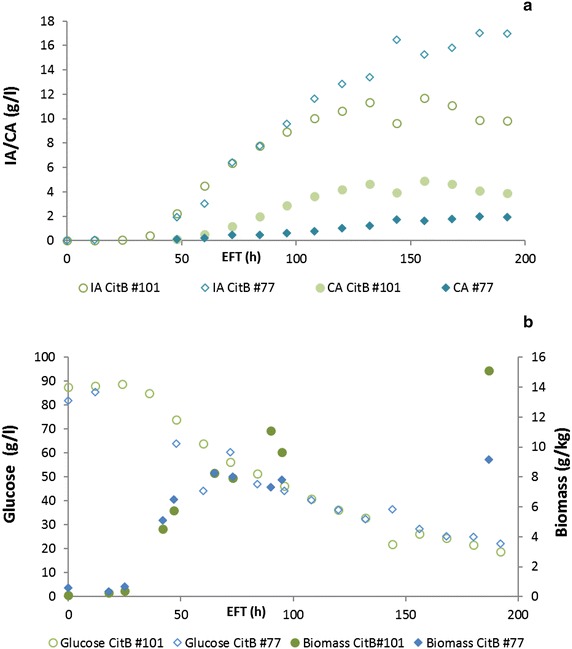
Controlled batch cultivations of *citB* overexpressing mutants. HPLC analysis of controlled batch cultivations of CitB #77 and #101. **a** Production of IA starts simultaneously for both strains after 36 h where CitB#77 ends with a final titer of 16.98 g/L IA after 192 h and CitB #101 reaches a final titer of 9.82 g/L. These two strains also produce CA, unlike the CitB #99 and #113 strains. CitB #77 produces a final titer of 1.9 g/L CA and CitB #101 produces 3.9 g/L CA. **b** Biomass development during controlled batch cultivations of CitB #77 and #101

Controlled batch-cultivations were performed with the best four *citB* transformants CitB#77, #99, #101 and #113, together with their parental strain AB1.13 #49. Maximum production of IA increased from 0.15 g/L/h for AB1.13 #49 to 0.35 g/L/h for AB1.13 CitB#99 and 0.26 g/L/h for CitB#113. CitB#99 and #113 reached final IA titers of 26.2 and 23.4 g/L compared to a final titer of 12.96 g/L for AB1.13 #49. Interestingly CitB#99 and #113 do not secrete CA whereas AB1.13 #49 secretes up to 8.91 g/L CA showing complete conversion of precursor CA to IA in CitB#99 and #113 (Fig. 7). The CitB #77 and CitB#101 strains, contrary to #99 and #113, still produce low amounts of CA with final titers of 1.96 g/L (#77) and 4.92 g/L (#101) (Fig. 8). Also the maximum production rate for IA is lower at 0.16 g/L/h (#77) and 0.11 g/L/h (#101). The maximum productivity of AB1.13 #49 is even higher than that of CitB #101. Interestingly, the three high IA producing strains CitB#77, #99 and #113 form less biomass during the course of the experiment than the less IA producing parental strain (#49) and CitB#101 (Figs. 7, 8). This observation highlights the potential antimicrobial effect of IA again in accordance to the results as shown in Fig. 4.

## Discussion

Organic acid production in filamentous fungi has historically been enigmatic, almost a black box with a very high efficiency [25]. In our research we have obtained new insight in a specific case, i.e. IA production in *Aspergillus niger*. As already suggested earlier [8, 9] we have further confirmed that the three genes of the IA gene cluster in *A. terreus* all contribute to the efficiency of IA production in the heterologous host *A. niger.* Expression of only the *mttA* and *mfsA* gene in a *cadA* expression parental strain resulted in increased levels of CA, whereas expression of only *mttA* also resulted in increased levels of* cis*-aconitic acid in the extracellular medium [26]. These results show that uncoordinated expression of these transporters result in secretion of* cis*-aconitic acid, possibly relieving cytosolic accumulation of unwanted IA pathway intermediates. In addition, a role for MttA in mitochondrial transport of (*cis*-)aconitic acid is suggested. Based on the fact that at least three distinct classes of transformants are observed, we obtained further confirmation that a correct balance between mitochondrial transport of precursor molecules and cytosolic export of IA is required to establish the most efficient pathway towards IA. Expressing all three genes in the apparently correct levels resulted in the highest titer of IA and reduced titers of CA (Fig. 2a, b; Table 3). In these selected transformants overexpressing the entire IA gene cluster we also observed reduced glucose consumption and reduced biomass formation of high IA producing strains during the end of the fermentation (Fig. 3). As shown in Fig. 4 this is probably due to growth inhibition by IA itself as in the presence of 10 g/L IA *A. niger* shows relevant growth inhibition. Obviously (weak) organic acid toxicity is a well-known phenomenon in inhibiting fungal growth [27]. The effect of IA production on growth of *A. niger* was further analysed using transcriptome analysis (Hossain et al. in prep).

Recently Chen et al. have elucidated a IA degradation pathway in the natural producer *A. terreus* that metabolizes IA into the building block chemicals acetyl-CoA and pyruvate [28]. This pathway bears remarkable similarity with the IA detoxification pathway identified in the pathogenic species *Pseudomonas aeruginosa* and *Yersinia pestis* by Sasikaran et al. [29]. Until now such IA detoxification pathway has not been characterized for *A. niger*, however, this will be further addressed in our ongoing research to improve IA levels.

**Fig. 9:**
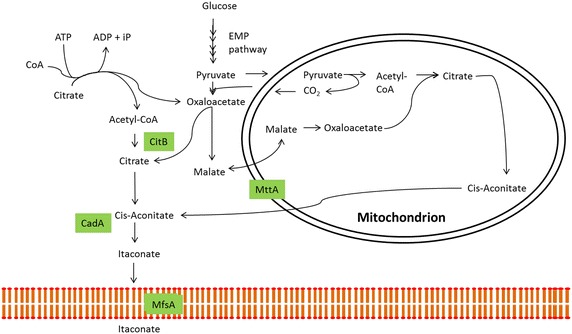
Hypothetical pathway of itaconic acid production in *citB* overexpressing *A. niger* mutants. Hypothetical pathway in *citB* overexpressing IA producing strains. CitB has no known mitochondrial targeting sequence and is thought to be located in the cytosol where it converts acetyl-CoA and oxaloacetate into citrate. Citrate continues to be further processed by aconitase into* cis*-aconitate which eventually is decarboxylated by CadA into IA. Theoretically,* cis*-aconitate shuttling from mitochondria to cytoplasm by MttA is redundant in this novel pathway. Note that only direct pathway steps towards IA production are shown

In a preliminary analysis of the expression profile of a selection of genes related to organic acid production (Additional file 1), we observed a very significant overexpression of two *A. niger* genes, one encoding a citrate synthase and one encoding a CAD-like protein. Both genes are members of a larger gene cluster encoding a secondary metabolite possibly related to IA (Palys et al. manuscript in preparation). Interestingly, in contrast to the canonical citrate synthase CitA this novel citrate synthase, now termed CitB, has no mitochondrial targeting sequence. In an attempt to improve IA production, Blumhoff et al. [30], addressed the aspect that the native pathway in *A. terreus* was considered to take place in two compartments, i.e. the mitochondrion (citrate synthase and aconitase) and the cytosol (*cis*-aconitate decarboxylase) [31]. Expression of the aconitase and* cis*-aconitate decarboxylase genes in either the mitochondrion or the cytosol resulted in improved IA levels in all cases [30]. The finding of a putative cytosolic citrate synthase allowed us to design a non-mitochondrial biosynthetic pathway by overexpression of the *citB* gene in a strain expressing the IA gene cluster (Fig. 9). As shown in Fig. 6, overexpression of *citB* resulted in significant increase in IA titers. Moreover, hardly any CA was produced in two of the *citB* overexpressing strains, further demonstrating the positive effect of *citB* overexpression on IA production. It may be somewhat counter intuitive to observe that the overexpression of a citrate synthase would lead to a reduction in CA titer. However, as we show, this reduction in CA titer is accompanied by an increase in IA titer. Therefore we assume that by overexpressing *citB* we have engineered a pathway that enables cytosolic synthesis of citrate and concomitantly its conversion into IA and thus preventing CA to be exported. As shown in Fig. 8 not in all *citB* transformants CA production is completely abolished. In controlled batch-cultivations with strains CitB #77 and #101 low levels of CA are still observed in the culture medium.

It should be noted that in the classical IA pathway also aconitate dehydratase (or aconitase) activity plays a role, however, none of the aconitate dehydratase-like genes from *A. niger,* apart from the canonical mitochondrial enzyme related to the TCA cycle, was significantly expressed (Additional file 1), raising the question how citrate is converted into* cis*-aconitate in the cytosol. Interestingly, however, members of the gene family encoding CAD like proteins have also been reported to encode citrate dehydratase activity [33]. Therefore we speculate that the *A. terreuscadA* gene and/or the gene encoding the CAD-like protein in the *citB* gene cluster are able to perform both citrate dehydratase and* cis*-aconitate decarboxylase activity, alleviating the need for a specific aconitate dehydratase protein. Our previous attempts to purify the *A. terreus* CadA protein, had already suggested the presence of a protein with both aconitase and* cis*-aconitate decarboxylase activity (our unpublished results). In this respect it is also interesting to note that, similar as the IA gene cluster, the *citB* gene cluster does not carry a aconitase encoding gene, while this type of activity would be expected to be required for the production of the pathway specific secondary metabolite [23]. Further biochemical analysis will be required to confirm if the CAD-like proteins in *A. niger* indeed fulfill both steps in the organic acid pathway. Obviously, the idea that CAD-like proteins would fulfill both steps would also be an alternative solution for preventing a futile cycle based on citrate synthase, aconitase and ATP-citrate lyase in the same compartment. This may be in particular relevant as recent results have revealed that the mitochondrial tricarboxylic acid transporter MttA, does not export citric acid from the mitochondrion to the cytosol [26]. Whether MttA and endogenous *A. niger* mitochondrial tricarboxylic acid transporters are still contributing to itaconic acid production in the presence of a cytosolic pathway is currently not known and goes beyond the topic of this paper.

Table 4 summarizes production of various IA producing *A.niger* strains engineered by us. A clear increase in titer, yield and productivity of IA is seen in *citB* overexpressing strains. Obviously the obtained levels are still below industrial target levels [32]. Therefore, studies to convert industrial CA producing *A. niger* strains into an industrial IA producing strain are underway. These strains can then be fermented under relevant industrial fermentation conditions to obtain the required IA productivity.

**Table 4:** An overview of controlled batch cultivations with IA producing *A. niger* strains

Strain	Highest titer g/L		Max productivity g/L/h		Yield g/g glucose		
	Itaconate	Citrate	Itaconate	Citrate	Itaconate	Citrate	Biomass (g/kg)
AB1.13 CAD 4.1	1.45	15.95	0.01	0.19	0.02	0.22	17.33
AB1.13 CAD + MFS 3.9	1.36	4.19	0.02	0.09	0.04	0.11	9.54
AB1.13 + CAD + MFS + MTT #08	2.48	21.99	0.02	0.13	0.03	0.29	15.59
AB1.13 + CAD + MFS + MTT #63	9.52	18.03	0.05	0.11	0.13	0.24	13.53
AB1.13 + CAD + MFS + MTT #49	11.74	13.17	0.08	0.12	0.16	0.18	15.02
AB1.13 + CAD + MFS + MTT #49	12.96	8.91	0.15	0.13	0.20	0.14	21.69
AB1.13 CitB#77	17.02	1.96	0.16	0.02	0.28	0.03	9.18
AB1.13 CitB#101	11.69	4.92	0.11	0.07	0.19	0.08	15.13
AB1.13 CitB#99	26.22	0.30	0.35	NA	0.37	NA	9.9
AB1.13 CitB#113	23.42	0.35	0.26	NA	0.33	NA	12.38

Overview of titer, productivity and yield of IA and CA during controlled batch-cultivations. Yield and biomass are measured at point of highest titer. In Fig. 9 an overview of the metabolic pathway consisting of the activities encoded by cadA, citB, mttA and mfsA is given

It should be noted that our newly designed *cadA*/*citB* IA pathway consists of biosynthetic steps derived from two unrelated (secondary metabolite) gene clusters from two different organisms. The IA gene cluster is lacking in *A. niger*, whereas the *citB* gene cluster is lacking in *A. terreus*. This type of metabolic rewiring and versatility in *Aspergillus* is not completely unprecedented, as very recently Gou et al. [34] revealed that genes from different metabolite gene clusters can result in partly overlapping biosynthetic pathways.

Interestingly, also a few other fungal species are known to produce IA [1, 35]. Among these *Ustilagomaydis* is the most well studied species. For this species also the complete genome sequence has been analyzed, allowing to perform genome mining for the presence of the orthologous IA gene cluster. Surprisingly, based on BLAST searches a gene cluster similar to the one observed in *A. terreus* is not present in *Ustilago*. However, recently Geiser et al. [24] have shown that in *Ustilago* IA is naturally produced from a completely different biosynthetic pathway using* trans*-aconitic acid as precursor, which is synthesized from* cis*-aconitate via a* cis*–*trans* aconitate isomerase. Similar as the IA biosynthetic pathway in *A. terreus* the *Ustilago* pathway is clustered in the genome together with a mitochondrial transporter and a major facilitator exporter and without any aconitase or aconitate dehydratase. However, none of these transporters are orthologous to those in the *A*. *terreus* gene cluster. Based on these results it is clear that the fungal pathways to a relatively simple metabolite like IA show a high level of diversity in different species. By linking parts of the different pathways together we have been able to create a highly efficient pathway with the smallest number of proteins using a two component citrate synthase and citrate dehydratase/*cis*-aconitate decarboxylase pathway.

One aspect that we have not yet addressed in our research is the origin of the substrate required for the cytosolic citrate synthase. In the mitochondrion the precursors for citrate synthase are oxaloacetate and acetyl-CoA. Oxaloacetate is produced from pyruvate by the action of cytosolic pyruvate carboxylase. There are different mechanisms by which acetyl-CoA can be synthesized in the cytosol [36, 37]. How these pathways, which are also largely compartmentalized, contribute to the IA production in our strains goes clearly beyond the topic of this paper and is subject of our ongoing research (Hossain et al. in preparation). In our ongoing research these more generic aspects of itaconic acid biosynthesis will be addressed [34, 35]. How these pathways, which are also largely compartmentalized, contribute to the IA production in our strains goes clearly beyond the topic of this paper and is subject of our ongoing research (Hossain et al. in preparation).

## Conclusions

Previously we have reported the stable expression of *cadA* in AB1.13 which resulted in low level IA production. Strains carrying a *cadA* + *mttA* and *cadA* + *mfsA* background performed better than strains carrying only *cadA*. Engineering of strains that carried the full cluster resulted in higher productivity, yield and titer of IA. However, our results showed that IA production has a negative growth effect on *A. niger*. In order to understand what effect the production of this novel metabolite could have on *A. niger* metabolism we performed an RNA-Seq analysis on shake flask grown cultures of AB1.13 WT and AB1.13 CAD. Interestingly, preliminary analysis of the RNA-Seq data showed induction of a putative cytosolic citrate synthase *citB* that belongs to a secondary metabolite cluster that is hypothesized to be involved in the production of alkylated derivatives of IA. We overexpressed *citB* in AB1.13 #49 strains which carries the full IA biosynthesis cluster and by doing so we have reached IA titers up to 26.2 g/L in controlled batch-cultivations at a max production rate of 0.35 g/L/h and no detection of side product formation. Studies to integrate this novel IA production pathway in an industrial CA producing *A. niger* strain with the aim to convert it into an industrial IA producing strain are underway.

## Additional file

10.1186/s12934-016-0527-2 Comparison of differentially annotated sequence of MfsA; **Table S1.** RNA-Seq data of genes involved in organic acid biosynthesis; **Table S2.** RNA-Seq data of secondary metabolite cluster that is upregulated in itaconic acid producing conditions; **Figure S2.** Protein sequence comparison of CitA and CitB from *Aspergillus niger*; **Table S3.** RNA-Seq data of known *prpD*-like genes in *Aspergillus niger*; **Table S4.** Orthologues of *tad1* and *adi1* of *Ustilago maydis* in *Aspergillus niger*.

## Abbreviations

CAD: *cis*-aconitate decarboxylase
MTT: mitochondrial tricarboxylate transporter
MFS: major facilitator superfamily
CITB: citrate synthase B
WT: wild type
IA: itaconic acid
CA: citric acid
