# Supplementary material for: Rewiring a secondary metabolite pathway towards itaconic acid production in Aspergillus niger
Source: Microb Cell Fact. 2016 Jul 28;15:130. doi: 10.1186/s12934-016-0527-2 (PMC4965889; doi:10.1186/s12934-016-0527-2)
Supplement: Supplementary file 1 — 10.1186/s12934-016-0527-2 Comparison of differentially annotated sequence of MfsA; Table S1. RNA-Seq data of genes involved in organic acid biosynthesis; Table S2. RNA-Seq data of secondary metabolite cluster that is upregulated in itaconic acid producing conditions; Figure S2. Protein sequence comparison of CitA and CitB from Aspergillus niger; Table S3. RNA-Seq data of known prpD-like genes in Aspergillus niger; Table S4. Orthologues of tad1 and adi1 of Ustilago maydis in Aspergillus niger. [file 12934_2016_527_MOESM1_ESM.docx]

**Additional file 1: Figure S1.**

**The differences of the various encoded protein sequences compared to the encoded protein sequence corresponding to the synthetic mfsA gene from Li et al., 2013 are indicated in yellow**

**Synthetic gene Li A, Pfelzer N, Zuijderwijk R, Brickwedde A, van Zeijl C, Punt P. 2013. Reduced by-product formation and modified oxygen availability improve itaconic acid production in Aspergillus niger. Appl Microbiol Biotechnol 97:3901–11**

**CDG56264 Van der Straat L, Tamayo-Ramos JA, Schonewille T, de Graaff LH. Overexpression of a modified 6-phosphofructo-1-kinase results in an increased itaconic acid productivity in Aspergillus niger. AMB Express. 2013;3:57. doi:10.1186/2191-0855-3-57.**

**AGV15467 Huang X, Lu X, Li Y, Li X, Li J-J. 2014. Improving itaconic acid production through genetic engineering of an industrial Aspergillus terreus strain. Microb Cell Fact 13:119.**

Synthetic gene MGHGDTESPNPTTTTEGSGQNEPEKKGRDIPLWRKCVITFVVSWMTLVVTFSSTCLL

CDG56264 MHGRGDTESPNPATTSEGSGQNEPEKKGRDIPLWRKCVITFVVSWMTLVVTFSSTCLL 4

AGV15467 MSHGDTESPNPVTSTEGSGQSEPEKRGPDIPLWRKCVITFVVSWMTLVVTFSSTCLL 6

Synthetic gene PAAPEIANEFDMTVETINISNAGVLVAMGYSSLIWGPMNKLVGRRTSYNLAISMLCACSA

CDG56264 PAAPEIANEFDMTVETINISNAGVLVAMGYSSLIWGPMNKLVGRRTSYNLAISMLCACSA

AGV15467 PAAPEIAGEFDMTVETINISNAGVLIAMGYSSLIWGPMNKLIGRRTSYNLAISMLCACSA 3

Synthetic gene GTAAAINEEMFIAFRVLSGLTGTSFMVSGQTVLADIFEPVYRGTAVGFFMAGTLSGPAIG

CDG56264 GTAAAINEKMFIAFRVLSGLTGTSFMVSGQTVLADIFEPVYRGTAVGFFMAGTLSGPAIA 2

AGV15467 GTAAAINEEMFIAFRVLSGLTGTSFMVSGQTVLADIFEPVYRGTAVGFFMAGTLSGPAIG

Synthetic gene PCVGGVIVTFTSWRVIFWLGLGMSGLGLVLSLLFFPKIEGNSEKVSTAFKPTTLVTIISK

CDG56264 -CVGGVIVTFTSWRVIFWLQLGMSGLGLVLSLLFFPKIEGTSEKVSTAFKPTTLVSIISK 3

AGV15467 PCVGGIIVTFTSWRVIFWLQLAMSGLGLVLSLLFFPKVEAPSEKASTASKPTTLVTIISK 7

Synthetic gene FSPTDVLKQWVYPNVFLA----------------------DLCCGLLAITQYSILTSARA

CDG56264 FSPTDVLKQWVYPNVFLAVSAWEICPLHLLETKCSCRKQKDLCCGLLAITQYSILTSARA

AGV15467 FSPTDVLKQWVYPNIFLA----------------------DLCCGLLAITQYSILTSARA 1

Synthetic gene IFNSRFHLTTALVSGLFYLAPGAGFLIGSLVGGKLSDRTVRRYIVKRGFRLPQDRLHSGL

CDG56264 IFNSRFHLTTALVSGLFYLAPGAGFLIGSLVGGKLSDRTVRRYIVKRGFRLPQDRLHSGL

AGV15467 VFNSRFHLTTALVSGLFYLAPGAGFLIGSLVGGKLSDRTVRSYIVKRGFRLPQDRLHSGL 2

Synthetic gene ITLFAVLPAGTLIYGWTLQEDKGDMVVPIIAAFFAGWGLMGSFNCLNTYVA-EALPRNRS

CDG56264 ITLFAVLPAGTLIYGWTLQEDKGGMVVPIIAAFFAGWGLMGSFNCLNTYVAVEALPRNRS 2

AGV15467 ITLFAVLPAGTLIYGWTLQEGKGGMVVPIIAAFFAGWGLMGSFNCLNTYVA-EALPQNRS 3

Synthetic gene AVIAGKYMIQYTFSAGSSALVVPVIDALGVGETFTLCVVASTIAGLITAAIARWGINMQR

CDG56264 AVIAGKYMIQYSFSAGSSALVVPVIDALGVGWTFTLCVVASTIAGLITAAIARWGINMQR 2

AGV15467 AVIAGKYMLQYSFSAGSSAVVDLVINALGVGWTFTLCVVASTIAGLITAAIARWGINMQR 6

Synthetic gene WAERAFNMPTQ

CDG56264 WAERAFNLPTQ 1

AGV15467 WAEKAFNMPTQ 1

**Additional file 1: Table S1.**

| **Gene ID** | **Enzyme** | **TargetP** | **Gene name** | **AB1.13 WT RPKM** | | **AB1.13 CAD RPKM** | | **2LogR** | **Fold induction** |
| --- | --- | --- | --- | --- | --- | --- | --- | --- | --- |
| ANI_1_1206064 | pyruvate dehydrogenase E1 component subunit alpha (*pda1)* | Mito | An07g09530 | 263,81 | 264,72 | 205,40 | 207,09 | -0,36 | 0,78 |
| ANI_1_622094 | pyruvate dehydrogenase E1 component subunit alpha | Mito | An11g04550 | 10,81 | 9,47 | 10,54 | 9,75 | 0,00 | 1,00 |
| ANI_1_12014 | pyruvate dehydrogenase E1 component subunit beta (*pdhC*) | Mito | An01g00100 | 215,18 | 213,19 | 177,40 | 176,43 | -0,27 | 0,83 |
| ANI_1_274064 | pyruvate dehydrogenase E2 component (*pdhA*) | Mito | An07g02180 | 299,81 | 301,04 | 286,65 | 284,54 | -0,07 | 0,95 |
| ANI_1_440184 | Pyruvate carboxylase (*pycA*) | Other | An04g02090 | 269,38 | 269,16 | 341,88 | 342,07 | 0,34 | 1,27 |
| ANI_1_936024 | pyruvate decarboxylase (*pdcA*) | Other | An02g06820 | 5910,75 | 5915,54 | 304,97 | 306,49 | -4,27 | 0,05 |
| ANI_1_876084 | citrate synthase (*citA*) | Mito | An09g06680 | 478,40 | 482,85 | 428,47 | 426,67 | -0,17 | 0,89 |
| ANI_1_1226134 | methylcitrate synthase (*mcsA*) | Mito | An15g01920 | 51,49 | 51,55 | 25,69 | 24,11 | -1,02 | 0,49 |
| ANI_1_1474074 | citrate synthase (*citB*) | Other | An08g10920 | 58,09 | 57,90 | 522,44 | 521,45 | 3,15 | 8,86 |
| ANI_1_2950014 | citrate synthase | Other | An01g09940 | 3,86 | 3,52 | 0,91 | 0,96 | -1,28 | 0,41 |
| N.A. | citrate synthase | Mito | An09g03570 | 0,00 | 0,00 | 0,00 | 0,00 | 0,00 | 0,00 |
| ANI_1_76094 | ATP-citrate lyase (*aclB*) | Other | An11g00510 | 325,61 | 325,45 | 506,32 | 505,54 | 0,63 | 1,55 |
| ANI_1_78094 | ATP-citrate lyase (*aclA*) | Other | An11g00530 | 331,20 | 332,23 | 587,40 | 587,02 | 0,82 | 1,77 |
| ANI_1_1952184 | homocitrate synthase | Other | An04g06210 | 86,32 | 84,16 | 55,15 | 52,84 | -0,65 | 0,64 |
| ANI_1_470084 | aconitate hydratase | Mito | An09g03870 | 64,88 | 64,41 | 26,01 | 26,07 | -1,28 | 0,41 |
| ANI_1_3018024 | aconitate hydratase | Other | An02g11040 | 0,04 | 0,04 | 0,00 | 0,00 | -0,06 | 0,96 |
| ANI_1_1410074 | aconitate hydratase (*acoA*) | Mito | An08g10530 | 234,09 | 234,83 | 397,94 | 397,68 | 0,76 | 1,69 |
| ANI_1_1808144 | aconitate hydratase | Other | An16g05760 | 0,85 | 0,85 | 0,98 | 0,99 | 0,10 | 1,07 |
| ANI_1_578044 | aconitase | Other | An05g02230 | 6,29 | 6,38 | 14,13 | 14,28 | 1,05 | 2,07 |
| ANI_1_1802134 | aconitase (*acoC*) | Other | An15g07730 | 26,63 | 26,21 | 30,62 | 30,72 | 0,21 | 1,15 |
| ANI_1_1106134 | homoaconitate hydratase (*lysF*) | Mito | An15g00350 | 42,52 | 41,77 | 24,01 | 24,37 | -0,78 | 0,58 |
| ANI_1_440024 | isopropylmalate isomerase (IPMI) (*luA*) | Other | An02g03250 | 41,09 | 40,63 | 26,58 | 26,36 | -0,61 | 0,66 |
| ANI_1_906164 | isocitrate dehydrogenase (NAD+) subunit 1 | Mito | An18g06760 | 330,00 | 330,65 | 235,04 | 235,97 | -0,49 | 0,71 |
| ANI_1_798074 | isocitrate dehydrogenase [NAD] subunit 2 | Mito | An08g05580 | 282,75 | 285,59 | 239,56 | 239,30 | -0,25 | 0,84 |
| ANI_1_3136024 | isocitrate dehydrogenase [NADP] (*idpA*) | Other | An02g12430 | 103,56 | 104,13 | 88,58 | 88,64 | -0,23 | 0,85 |
| ANI_1_826184 | 2-oxoglutarate dehydrogenase (*kgdA*) | Mito | An04g04750 | 200,30 | 199,49 | 178,12 | 178,23 | -0,17 | 0,89 |
| ANI_1_1482094 | dihydrolipoyllysine-residue succinyltransferase (*kgdB*) | Mito | An11g11280 | 127,65 | 129,00 | 117,63 | 118,13 | -0,12 | 0,92 |
| ANI_1_230154 | succinyl-CoA ligase [GDP-forming] subunit alpha | Mito | An17g01670 | 133,28 | 132,83 | 147,85 | 148,10 | 0,15 | 1,11 |
| ANI_1_58124 | succinyl-CoA ligase [GDP-forming] subunit beta | Mito | An14g00310 | 178,16 | 181,01 | 173,87 | 174,14 | -0,05 | 0,97 |
| ANI_1_1750024 | succinate dehydrogenase [ubiquinone] flavoprotein subunit | Mito | An02g12770 | 77,25 | 77,97 | 48,65 | 48,60 | -0,66 | 0,63 |
| ANI_1_2706024 | succinate dehydrogenase [ubiquinone] flavoprotein subunit | Mito | An02g07600 | 0,24 | 0,29 | 0,51 | 0,47 | 0,24 | 1,18 |
| ANI_1_952104 | Fumarate hydratase (*fumR*) | Mito | An12g07850 | 203,87 | 201,12 | 130,25 | 129,88 | -0,63 | 0,64 |
| ANI_1_12134 | Malate dehydrogenase (*mdhA*) | Other | An15g00070 | 348,62 | 349,46 | 310,80 | 310,95 | -0,17 | 0,89 |
| ANI_1_12134 | Malate dehydrogenase (*mdh1*) | Mito | An07g02160 | 621,37 | 623,86 | 524,94 | 527,05 | -0,24 | 0,85 |
| ANI_1_2230094 | Malate dehydrogenase (*mdhB*) | Other | An11g07190 | 0,17 | 0,09 | 0,00 | 0,00 | -0,17 | 0,89 |
| ANI_1_2114184 | L-lactate dehydrogenase (*ldhA*) | Mito | An04g08220 | 0,00 | 0,00 | 0,00 | 0,00 | 0,00 | 1,00 |
| ANI_1_92174 | oxaloacetate acetylhydrolase (*oahA*) | Other | An10g00820 | 6,22 | 5,94 | 4,58 | 4,55 | -0,35 | 0,79 |
| ANI_1_2054064 | oxaloacetate acetylhydrolase (*dmmL*) | Mito | An07g08390 | 17,87 | 17,90 | 5,19 | 5,37 | -1,59 | 0,33 |
| ANI_1_1800134 | oxaloacetate hydrolase class protein | Other | An15g07720 | 50,04 | 48,68 | 47,56 | 49,92 | -0,02 | 0,99 |
| ANI_1_1256014 | methylisocitrate lyase (*mclA*) | Other | An12g07630 | 49,67 | 50,08 | 30,26 | 30,05 | -0,71 | 0,61 |
| ANI_1_1256014 | isocitrate lyase (*acuB*) | Other | An01g09270 | 23,20 | 23,04 | 9,36 | 9,15 | -1,23 | 0,43 |
| ANI_1_1336134 | isocitrate lyase/malate synthase | Other | An15g02980 | 9,07 | 9,21 | 7,59 | 8,13 | -0,19 | 0,87 |
| ANI_1_1826104 | isocitrate lyase/malate synthase | Other | An12g05180 | 0,28 | 0,28 | 0,00 | 0,00 | -0,36 | 0,78 |
| ANI_1_320134 | malate synthase (*acuE*) | Other | An15g01860 | 86,63 | 84,43 | 27,12 | 27,35 | -1,62 | 0,33 |

**Additional file 1: Table S2.**

| **Gene ID** | **Enzyme** | **Gene name** | **AB1.13 WT RPKM** | | **AB1.13 CAD RPKM** | | **2LogR** | **Fold induction** |
| --- | --- | --- | --- | --- | --- | --- | --- | --- |
| ANI_1_2488074 | sterigmatocystin biosynthesis fatty acid synthase subunit beta | An08g10860 | 35,78 | 35,72 | 386,76 | 384,34 | 3,39 | 10,52 |
| ANI_1_2490074 | prpD 2-methylcitrate dehydratase | An08g10870 | 48,70 | 51,13 | 475,19 | 474,40 | 3,22 | 9,35 |
| ANI_1_2492074 | GAL4; GAL4-like Zn2Cys6 binuclear cluster DNA-binding domain; found in transcription regulators like GAL4 | An08g10880 | 7,72 | 7,71 | 159,05 | 160,07 | 4,20 | 18,43 |
| ANI_1_1474074 | citrate synthase | An08g10920 | 58,09 | 57,90 | 522,44 | 521,45 | 3,15 | 8,86 |
| ANI_1_2494074 | elong_cond_enzymes | An08g10930 | 47,02 | 46,89 | 740,54 | 739,75 | 3,95 | 15,46 |
| ANI_1_2500074 | MFS multidrug transporter | An08g10970 | 153,73 | 154,14 | 721,08 | 718,65 | 2,22 | 4,65 |

**Additional file 1: Figure S2.**

citA MAST----LRLGTSALRSTSIAAKPVVQSAAFNGLRCYSTG------KAKSLKETFAEKL 50

citB MPDIASNGARNGASQNA-ETKPEPPVLHVVDSRTGKYFPIPIVRNAINASEFKKLKSPED 59

* . * *:* : **:: . . : : :*..:*: : :

citA PAEIEKVKKLRKEHGSKVIGEVTLDQAYGGARGVKCLVWEGSVLDSEEGIRFRGRTIPEC 110

citB PAHP----EDQNEQGIR-----VFDPGYSNTAVSESQV--TYIDGLKGTIQYRGYNIEDI 108

**. : ::*:* : .:* .*. : :. * : : *::** .* :

citA QELLPKAPGGQEPLPEGLFWLLLTGEIPTEQQVRDLSAEWAARSDLPKFIEELIDRCPST 170

citB V-------G-KKKFI-DTAHLLIWGEWPTPEQAKSLQEKLSSVPVLDESVFKVIQAFPPN 159

* :: : **: ** ** :*.:.*. : :: * : : ::*: * .

citA LHPMSQFSLAVTALEHESAFAKAYAKGINKKDYWNY---TFEDSMDLIAKLPTIAAKIYR 227

citB SSIIGMMIAALSAVQST-QMDRI-PAHAAKNLYLGNPKAVDDEIVRLMGSLSMITAAVYC 217

:. : *::*:: : : *: * . :: : *:..* *:* :*

citA NVFKDGKVAPIQKDKDYSYNLANQLGYG------DNNDFVE-LMRLYLTIHSDHEGGNVS 280

citB HHTG-REFTPPRPELSYIENFLLMMGHVESSTGLPNPQYVDRIERLWVLI-ADHEMTCST 275

. :.:* : : .* *: :*: * ::*: : **:: * :*** :

citA AHTTHLVGSALSSPMLSLAAGLNGLAGPLHGLANQEVLNWLTKMKAAIGNDLSDEAIKNY 340

citB A-AFLQTASSLPDVFSCMISALSALYGPLHGGAIEVAYKNFEEIGSV-------ENVAAK 327

* : ..*:* . : .: :.*..* ***** * : . : : :: :. * :

citA LWSTLNAGQVVPGYGHAVLRKTDPRYVSQREFALR---KLPDDPMFKLVSQVYKIAPGVL 397

citB IERVKAGKERLYGYGHRIYRVTDPRFIFIRQILDELKEEIARNPLLKVAFEVDRVASED- 386

: . . : : **** : * ****:: *:: . :: :*::*:. :* ::*

citA TEHGKTKNPYPNVDAHSGVLLQYYGLTEANYYTVLFGVSRALGVLPQLIIDRALGAPIER 457

citB -EYFVTRKLRPNADLFAALVYSAMGFPTE-FILPLSLLSRTQGFMAHWKEAMSSTARIWR 444

*: *:: **.* .:.:: . *: : * :**: *.: : : * * *

citA PKSYSTEAFAKLVGAKL 474

citB PGQIYTGHLNREMA--- 458

* . * : : :.

**Additional file 1: Table S3.**

| **Gene ID** | **Enzyme** | **TargetP** | **Gene name** | | **AB1.13 WT RPKM** | | **AB1.13 CAD RPKM** | | **2LogR** | **Fold induction** |
| --- | --- | --- | --- | --- | --- | --- | --- | --- | --- | --- |
| ANI_1_2490074 | prpD 2-methylcitrate dehydratase | Other | | An08g10870 | 48,70 | 51,13 | 475,19 | 474,40 | 3,22 | 9,35 |
| ANI_1_306134 | prpD 2-methylcitrate dehydratase (*mcdB*) | Mito | | An15g01780 | 99,57 | 98,08 | 83,29 | 84,95 | -0,23 | 0,85 |
| ANI_1_2952014 | prpD | Other | | An01g09950 | 5,60 | 5,43 | 3,53 | 3,55 | -0,52 | 0,70 |
| ANI_1_1536084 | prpD | Other | | An09g06220 | 26,69 | 25,93 | 4,63 | 4,60 | -2,28 | 0,21 |
| ANI_1_3352024 | prpD (*mcdA*) | Other | | An02g14730 | 3,90 | 4,00 | 3,51 | 3,87 | -0,08 | 0,95 |
| ANI_1_2948014 | prpD 2-methylcitrate dehydratase | Other | | An01g09930 | 3,51 | 3,46 | 0,89 | 0,83 | -1,27 | 0,41 |

**Additional file 1: Table S4.**

| **Gene ID** | **Enzyme** | **TargetP** | **Gene name** | **AB1.13 WT RPKM** | | **AB1.13 CAD RPKM** | | **2LogR** | **Fold induction** |
| --- | --- | --- | --- | --- | --- | --- | --- | --- | --- |
| ANI_1_1132124 | argininosuccinate lyase/tad1 like | Other | An14g01340 | 0,07 | 0,07 | 0,26 | 0,26 | 0,23 | 1,17 |
| ANI_1_376014 | argininosuccinate lyase/tad1 like | Mito | An01g02970 | 3,33 | 3,20 | 10,04 | 10,10 | 1,38 | 2,60 |
| ANI_1_924164 | hypothetical protein | Other | An18g00050 | 5,34 | 5,53 | 4,72 | 4,60 | -0,19 | 0,88 |
| ANI_1_3022024 | hypothetical protein | Mito | An02g11060 | 2,06 | 2,17 | 1,91 | 2,08 | -0,06 | 0,96 |
| ANI_1_618114 | hypothetical protein | Mito | An13g01480 | 6,69 | 6,46 | 7,35 | 7,80 | 0,18 | 1,13 |
| ANI_1_1868104 | hypothetical protein | Other | An12g05470 | 0,25 | 0,26 | 0,11 | 0,11 | -0,17 | 0,89 |
